# Positioning-dependent bidirectional NELL2 signaling in the brain

**DOI:** 10.3389/fendo.2022.1049595

**Published:** 2022-10-18

**Authors:** Byung Ju Lee, Jin Kwon Jeong

**Affiliations:** ^1^ Department of Biological Sciences, College of Natural Sciences, University of Ulsan, Ulsan, South Korea; ^2^ Department of Pharmacology and Physiology, School of Medicine & Health Sciences, The George Washington University, Washington, DC, United States

**Keywords:** NELL2, neurotrophic peptide, endoplasmic reticulum, protein kinase C (PKC), cardiometabolism

## Introduction

A network with appropriate quality and quantity of intercellular and intracellular communication is fundamental for healthy brain function. In other words, molecular, cellular, and structural impairments of brain cells are associated with a broad range of pathophysiological conditions, including aging and age-associated diseases, motor and behavioral symptoms, and metabolic syndromes. A neural tissue-specific epidermal growth factor (EGF)-like protein was first identified in 1995 in chick embryos and named NEL (1). Subsequently, two mammalian homologs of chicken NEL were identified in rodents and humans and named NELL1 and NELL2 (2, 3). Among them, NELL2 is more closely related to NEL and is highly conserved among species including humans. An expression profile of NELL2 in rodent brains revealed the broad distribution of NELL2 throughout the entire brain (4). Interestingly, accumulating evidence has demonstrated that NELL2 is a secreting neuropeptide (2, 3, 5) and, at the same time, is an intracellular neuromodulator through a direct interaction with protein kinase C (PKC) within neurons (6, 7). Importantly, these investigations have further indicated a positioning-dependent conflicting outcome of NELL2-triggered signaling in downstream target gene activation within neurons (7) as well as in competitive synaptic remodeling between neurons (8, 9). Therefore, this review will discuss a site-specific multifactorial nature of NELL2 action in brain, and further a possible involvement of NELL2 in central cardiometabolism regulation.

## A site of NELL2 action: Intracellular vs. extracellular

In the beginning, investigations have mainly focused to characterize the biochemical and structural signature of NELL2 protein (2, 3), and revealed that NELL2 peptide consists of multiple functional domains, including the von Willebrand factor C, a thrombospondin-1-like structure, and several EGF-like repeats. In particular, some of the EGF-repeat domains possessed Ca^2+^-binding ability. The physiological function of these domains remains largely unidentified; however, accumulated investigations have recognized that these domains share common characteristics with extracellular matrix proteins known to be critical in neural growth and development (2, 3, 10). Indeed, NELL2 is a glycosylated protein and possesses a signal peptide domain that is necessary for secreting proteins (3), and is able to bind to the roundabout (Robo) family of membrane receptors (9, 11, 12), or an orphan receptor tyrosine kinase, c-ros oncogene 1 (ROS1) (13). Within the cytoplasm, localization of NELL2 was highly limited to the endoplasmic reticulum (ER), Golgi apparatus, and moving vesicles between soma and axons (5). These investigations together clearly indicate the nature of NELL2 as a secreting neuropeptide.

On the other hand, other studies have recognized an intracellular NELL2 action through the PKC–ERK pathway. In these studies, NELL2 was identified as a PKC-binding intracellular molecule through the EGF-like motifs (6), and an ablation of endogenous NELL2 synthesis reduced the survival of neurons under cell death conditions (14), and resulted in the shortening axonal projections of cells (8), both of which through the downregulation of ERK signaling. Based on these studies, it is reasonable to mention that NELL2 is a novel and unique molecule that can trigger both intracellular and extracellular signaling pathways simultaneously. Additionally, the EGF-like motifs on NELL2 might be the key to induce NELL2-dependent signaling cascades, regardless of the area of NELL2 action.

## Positioning-dependent contrasting NELL2 signaling

Our recent findings newly recognized a NELL2 function on gene expression of preproenkephalin (PPE) (7), a precursor peptide for multiple endogenous opioids that have been known to be involved in diverse brain functions including pain, stress, and cardiovascular and metabolic regulation (15–19). In this study, we first observed a negative regulatory role of intracellular NELL2 on PPE gene expression. Before being released, endogenously biosynthesized NELL2 would be processed through the ER as mentioned above (5), and therefore, an expression vector carrying a NELL2-coding region conjugated with an ER retention motif has been utilized to confine NELL2 in the ER. Importantly, accumulated NELL2 in the ER resulted in a reduced PPE gene expression through the Ca^2+^-binding EGF motifs, by downregulating a series of intracellular signaling pathways including PKC, ERK, and c-Fos. Surprisingly, the overall outcomes were opposite from the extracellular NELL2: a level of PKC, ERK, and c-Fos signaling is upregulated following extracellular NELL2 treatment, and thus, gene expression of PPE is enhanced. Additionally, the intracellular NELL2 action seemed to be dominant to extracellular NELL2 signaling in terms of PPE gene expression as an *in vivo* disruption of NELL2 synthesis resulted in an enhanced PPE gene expression in the rat brains. These observations clearly indicate a positioning-dependent bidirectional NELL2 function on PPE gene expression.

Interestingly, recent studies have also demonstrated a role for NELL2 in competitive synaptic remodeling in neurons (8, 9). Both extracellular and intracellular NELL2 induced overall axonal elongation of neurons (8), and therefore, NELL2 is suggested to be an activator of axonal projections regardless of the site of action. However, another investigation has further demonstrated that cells that received extracellular NELL2 from a certain direction withhold their fibers from the cell surface facing the NELL2, but elongate their fibers to the opposing direction (9). Therefore, the nature of extracellular NELL2 on axonal growth is likely inhibitory. Together, these results indicate a positioning-dependent opposing effect of NELL2 on PPE gene expression as well as synaptic remodeling.

## A possible role of NELL2 in central cardiometabolism regulation

As mentioned above, qualitative and quantitative information on NELL2-dependent molecular and cellular mechanisms is now available. However, a physiological NELL2 function has not been well determined in adult mammalian brains. Interestingly, our anatomical approaches to determining brain distribution of NELL2 revealed a relatively high expression of NELL2 in terms of both mRNA and protein levels in multiple cardiometabolic nuclei, including the subfornical organ (SFO), paraventricular and ventromedial hypothalamic nucleus (PVN and VMH, respectively), and the arcuate nucleus (ARC) in adult rodent brains (4, 20). Especially in the ARC, NELL2 expression is detected on proopiomelanocortin (POMC) cells as well as cells expressing neuropeptide Y (NPY) (20). In the context of central cardiometabolism regulation, the SFO and ARC are believed to be the forebrain gates for circulating cardiometabolic signaling molecules, and transform the information into the deep brain regions, such as the PVN, to regulate neuroendocrine and autonomic outcomes for the preservation of cardiometabolic homeostasis (21, 22). Following these anatomical observations, studies utilizing the loss-of-function approach further demonstrated an involvement of NELL2 in metabolism regulation (20). Hypothalamus-targeted ablation of NELL2 biosynthesis in adult rats resulted in a reduction in daily food intake and body weight gain. In addition, mRNA levels of hypothalamic NELL2 were increased under fasting conditions compared to those in a fed state. These molecular and behavioral results clearly indicate an involvement of hypothalamic NELL2 in metabolic homeostasis as an orexigenic molecule. Unlike PPE, NELL2 did not affect the gene expression of both POMC and NPY in the ARC. Therefore, an investigation aimed at understanding the detailed underlying mechanism through which NELL2 affects metabolism regulation is necessary.

## Conclusion

NELL2 is a secreting neuropeptide and thus believed to be an extracellular signaling molecule with an autocrine, paracrine, and/or endocrine nature. At the same time, NELL2 also possesses the ability to modify ER-initiated intracellular signaling pathways. A striking finding from us and other groups is that the outcome of NELL2 signaling is dependent on the site of NELL2 action: the effects of intracellular NELL2 signaling in terms of downstream gene expression and/or synaptic reorganization could be reversed by the NELL2 signaling initiated extracellularly.

In spite of recent efforts, NELL2-mediated cellular signaling and physiological functions are still largely unknown. For example, NELL2 is able to bind to certain receptors as described above; however, specific receptor-dependent NELL2 signaling has not been elucidated. Additionally, there is a possibility that some cells utilize NELL2 only as an intracellular modulator while the other cells use it exclusively as an extracellular molecule. Therefore, in-depth and targeted investigations to address these questions are clearly necessary.

NELL2 is a newly identified rising molecule in the field of brain physiology, and the site of NELL2 action should be considered in future studies.

## Author contributions

BL and JJ equally contributed to the design and development of the draft, and approved the final manuscript.

## Funding

This research was supported by the Priority Research Centers Program (2014R1A6A1030318) through the National Research Foundation of Korea (to BL).

## Conflict of interest

The authors declare that the research was conducted in the absence of any commercial or financial relationships that could be construed as a potential conflict of interest.

## Publisher’s note

All claims expressed in this article are solely those of the authors and do not necessarily represent those of their affiliated organizations, or those of the publisher, the editors and the reviewers. Any product that may be evaluated in this article, or claim that may be made by its manufacturer, is not guaranteed or endorsed by the publisher.

## References

[1] Matsuhashi S Noji S Koyama E Myokai F Ohuchi H Taniguchi S. New gene, nel, encoding a m(r) 93 K protein with EGF-like repeats is strongly expressed in neural tissues of early stage chick embryos. Dev Dyn (1995) 203(2):212–22. doi: 10.1002/aja.1002030209 7655083

[2] Watanabe TK Katagiri T Suzuki M Shimizu F Fujiwara T Kanemoto N. Cloning and characterization of two novel human cDNAs (NELL1 and NELL2) encoding proteins with six EGF-like repeats. Genomics (1996) 38(3):273–6. doi: 10.1006/geno.1996.0628 8975702

[3] Kuroda S Oyasu M Kawakami M Kanayama N Tanizawa K Saito N. Biochemical characterization and expression analysis of neural thrombospondin-1-like proteins NELL1 and NELL2. Biochem Biophys Res Commun (1999) 265(1):79–86. doi: 10.1006/bbrc.1999.1638 10548494

[4] Jeong JK Kim HR Hwang SM Park JW Lee BJ. Region- and neuronal phenotype-specific expression of NELL2 in the adult rat brain. Mol Cells (2008) 26(2):186–92. 18677093

[5] Ha CM Hwang EM Kim E Lee DY Chang S Lee BJ. The molecular mechanism of NELL2 movement and secretion in hippocampal progenitor HiB5 cells. Mol Cells (2013) 36(6):527–33. doi: 10.1007/s10059-013-0216-5 PMC3887960 24352699

[6] Kuroda S Tanizawa K. Involvement of epidermal growth factor-like domain of NELL proteins in the novel protein-protein interaction with protein kinase c. Biochem Biophys Res Commun (1999) 265(3):752–7. doi: 10.1006/bbrc.1999.1753 10600492

[7] Ha CM Kim DH Lee TH Kim HR Choi J Kim Y. Transcriptional regulatory role of NELL2 in preproenkephalin gene expression. Mol Cells (2022) 45(8):537–49. doi: 10.14348/molcells.2022.2051 PMC9385569 35950455

[8] Kim HR Kim DH An JY Kang D Park JW Hwang EM. NELL2 function in axon development of hippocampal neurons. Mol Cells (2020) 43(6):581–9. 10.14348/molcells.2020.0032 PMC7332358 32597395

[9] Jaworski A Tom I Tong RK Gildea HK Koch AW Gonzalez LC. Operational redundancy in axon guidance through the multifunctional receptor Robo3 and its ligand NELL2. Science (2015) 350(6263):961–5. doi: 10.1126/science.aad2615 26586761

[10] Nakamura R Nakamoto C Obama H Durward E Nakamoto M. Structure-function analysis of nel, a thrombospondin-1-like glycoprotein involved in neural development and functions. J Biol Chem (2012) 287(5):3282–91. doi: 10.1074/jbc.M111.281485 PMC3270983 22157752

[11] Pak JS DeLoughery ZJ Wang J Acharya N Park Y Jaworski A. NELL2-Robo3 complex structure reveals mechanisms of receptor activation for axon guidance. Nat Commun (2020) 11(1):1489. doi: 10.1038/s41467-020-15211-1 32198364 PMC7083938

[12] Yamamoto N Kashiwagi M Ishihara M Kojima T Maturana AD Kuroda S. Robo2 contains a cryptic binding site for neural EGFL-like (NELL) protein 1/2. J Biol Chem (2019) 294(12):4693–703. doi: 10.1074/jbc.RA118.005819 PMC6433047 30700556

[13] Kiyozumi D Noda T Yamaguchi R Tobita T Matsumura T Shimada K. NELL2-mediated lumicrine signaling through OVCH2 is required for male fertility. Science (2020) 368(6495):1132–5. doi: 10.1126/science.aay5134 PMC7396227 32499443

[14] Choi EJ Kim DH Kim JG Kim DY Kim JD Seol OJ. Estrogen-dependent transcription of the NEL-like 2 (NELL2) gene and its role in protection from cell death. J Biol Chem (2010) 285(32):25074–84. doi: 10.1074/jbc.M110.100545 PMC2915743 20538601

[15] Bowman BR Goodchild AK. GABA and enkephalin tonically alter sympathetic outflows in the rat spinal cord. Auton Neurosci (2015) 193:84–91. doi: 10.1016/j.autneu.2015.08.006 26329875

[16] Ikeda H Ardianto C Yonemochi N Yang L Ohashi T Ikegami M. Inhibition of opioid systems in the hypothalamus as well as the mesolimbic area suppresses feeding behavior of mice. Neuroscience (2015) 311:9–21. doi: 10.1016/j.neuroscience.2015.10.002 26454026

[17] Will MJ Vanderheyden WM Kelley AE. Striatal opioid peptide gene expression differentially tracks short-term satiety but does not vary with negative energy balance in a manner opposite to hypothalamic NPY. Am J Physiol Regul Integr Comp Physiol (2007) 292(1):R217–26. doi: 10.1152/ajpregu.00852.2005 16931647

[18] Yang J Zhang L Xie P Pan M Ma G. Transplantation of mesenchymal stromal cells expressing the human preproenkephalin gene can relieve pain in a rat model of neuropathic pain. Neurochem Res (2020) 45(9):2065–71. doi: 10.1007/s11064-020-03068-1 32529390

[19] Melo I Drews E Zimmer A Bilkei-Gorzo A. Enkephalin knockout male mice are resistant to chronic mild stress. Genes Brain Behav (2014) 13(6):550–8. doi: 10.1111/gbb.12139 24804898

[20] Jeong JK Kim JG Kim HR Lee TH Park JW Lee BJ. A role of central NELL2 in the regulation of feeding behavior in rats. Mol Cells (2017) 40(3):186–94. 10.14348/molcells.2017.2278 PMC5386956 28301916

[21] Jeong JK Dow SA Young CN. Sensory circumventricular organs, neuroendocrine control, and metabolic regulation. Metabolites (2021) 11(8):494. doi: 10.3390/metabo11080494 34436435 PMC8402088

[22] Jeong JK Kim JG Lee BJ. Participation of the central melanocortin system in metabolic regulation and energy homeostasis. Cell Mol Life Sci (2014) 71(19):3799–809. doi: 10.1007/s00018-014-1650-z PMC11113577 24894870

